# Active Disturbance Rejection Position Synchronous Control of Dual-Hydraulic Actuators with Unknown Dead-Zones

**DOI:** 10.3390/s20216124

**Published:** 2020-10-28

**Authors:** Lixin Wang, Dingxuan Zhao, Fucai Liu, Qian Liu, Zhuxin Zhang

**Affiliations:** 1 School of Mechanical Engineering, Yanshan University, Qinhuangdao 066004, China; wlx@stumail.ysu.edu.cn (L.W.); lq_ysu@stumail.ysu.edu.cn (Q.L.); zhzhxn@ysu.edu.cn (Z.Z.); 2 Key Laboratory of Special Carrier Equipment of Hebei province, Yanshan University, Qinhuangdao 066004, China; 3 School of Electrical Engineering, Yanshan University, Qinhuangdao 066004, China; lfc@ysu.edu.cn

**Keywords:** electro-hydraulic proportional system, position synchronous control, adaptive dead-zone inverse, active disturbance rejection control

## Abstract

In this paper, an integrated control strategy of position synchronization control for dual-electro-hydraulic actuators with unknown dead-zones is proposed. The unified control scheme consists of two parts: One is adaptive dead-zone inverse controllers of each hydraulic actuator to offset the unknown dead-zones. The other is the linear active disturbance rejection controller (LADRC) for position synchronization error. First, the model of the electro-hydraulic proportional position control system (EPPS) was identified by the forgetting factor recursive least square (FFRLS) algorithm. Next, the model reference dead-zone inverse adaptive controller (MRDIAC) was developed to compensate for the delay of actuator response caused by unknown proportional valve dead-zones. Meanwhile, the validity of the adaptive law was proven by the Lyapunov theory. Therefore, the position control accuracy of each hydraulic actuator is guaranteed. Besides, to improve the precision of position synchronization control of dual-hydraulic actuators, a simple and elegant synchronous error-based LADRC was adopted, which applies the total disturbances design concept to eliminate and compensate for motion coupling rather than cross-coupling technology. The performance of the proposed control solution was investigated through extensive comparative experiments based on a hydraulic test platform. The experimental results successfully demonstrate the effectiveness and practicality of the proposed method.

## 1. Introduction

Electro-hydraulic drive systems (EHDS) are widely used in industrial applications because of their high power-to-weight ratios, fast responses, and high stiffness holding capacities [1]. Moreover, the electro-hydraulic proportional system (EHPS) reduces the level of required oil filtration and is more cost-effective than an electro-hydraulic servo system (EHSS) [2]. As a consequence, it is becoming a crucial component of robotic excavators [3], shield tunneling machines [4], and hydraulic elevators [5]. Parallel mechanisms can perform more complex operations in restrained space and implement more powerful output forces/torques [6]. In particular, hydraulic parallel mechanisms play an essential role in heavy machinery [7]. However, the position control of the electro-hydraulic proportional hydraulic parallel mechanism faces two challenges. The first is axis synchronization, one of the issues that almost all parallel devices will encounter [8]. The second challenge involves the parameter uncertainty [9], unknown dead-zones [10], and unknown external disturbances of the EHPS [11]. These will lead to the control system to fail. In this paper, the position synchronization system of proportional valve-control dual-hydraulic actuators with unknown dead-zones is investigated.

Over the past few decades, many scholars have concentrated on synchronization control and developed various control methods. The proportional-integral-derivative (PID) controller is widely used in industrial automation applications. A low non-synchronous error is guaranteed by applying a constrained step PD controller in a twin-cylinder hydraulic elevator [5]. Motion coupling is an essential obstacle in synchronous control, so the decoupling control method is proposed [7]. Furthermore, dual closed-loop control is also an effective strategy to solve synchronous control problems. In [12], a control strategy consisting of an inner-loop controller and an outer-loop synchronous controller was designed to handle the nonlinearities and synchronization errors of the lifting cylinders, respectively. Similarly, to improve the synchronous position precision of the multi-axis rotating system, an acceleration controller was adopted in an inner loop to ensure the robustness of the speed of each axis against disturbances, and an outer-loop error comparison method has been introduced to minimize synchronous position errors [13]. Furthermore, modern control theories have provided many efficient control methods, such as robust control (RC), sliding mode control (SMC), and adaptive control (AC). A novel dynamic decoupling-based robust synchronous control scheme is proposed to solve the high-precision motion trajectory tracking control of a parallel hydraulic manipulator with the matching uncertainties and unmatched uncertainties [14]. The control strategy, including a global sliding mode control method and the cross-coupling technology, was introduced to achieve high-precision motion for a multi-axis servo system [15]. In order to realize the synchronization motion between cables, adding two synchronization controllers in the cable space was proposed to solve the effect of cable tension during the motion control of cable-driven parallel robots [16]. Intelligent control methods (ICMs), which have strong nonlinear approximation ability and operate like the human brain, are also employed to enhance the synchronization control system’s performance; examples include fuzzy control [17], fuzzy neural network control [18], and iterative learning control (ILC) [19]. Although these techniques have successfully improved the position synchronization control performance of each system, direct or indirect full state feedback and model information are required in almost all modern control theory methods. At the same time, abundant prior knowledge is needed in ICMs. Unfortunately, these requirements are sometimes not wholly met because of limitations imposed by installation space, cost, and operational experience.

Due to the limitations of the machining accuracy and solenoid performance, a high deadband generally exists in the proportional valve [20]. The parameters of the proportional valve dead-zone (DZ) are affected by spool wear, oil characteristics, and working pressure [21]. Therefore, adequate compensation for a dead-zone is the key to improving the EHPS’s position tracking accuracy. Considering the characteristics of the proportional valve, a dead-zone compensation method based on microflow rate detection was proposed [22]. Moreover, control algorithms are more commonly used to solve the dead-zone problem. Adaptive control (AC) is a powerful tool to handle time-varying parameters. An adaptive dead-zone inverse compensation controller was integrated to solve the problem of position tracking of a plant with an unknown dead-zone [23,24,25]. The robust adaptive control (RAC) method combines the advantages of adaptive control (AC) and robust control (RC). It is utilized to solve the problem of the actuator with an unknown dead-zone [26,27]. Besides, intelligent control methods such as fuzzy control [28] and neural network control [29] also have been extensively investigated in addressing the issue of the unknown dead-zone.

The active disturbance rejection controller (ADRC) is a relatively new control technology proposed by Han [30]. The essence of ADRC is the concept of “total disturbances,” and its estimation method—extended state observer (ESO). The “total disturbance” includes both internal unmodeled dynamics and unknown external disturbances. The plant is simplified to an approximate integrator chain structure, which is very easy to control by real-time estimation and compensation of ESO [31]. Therefore, ADRC offers a solution where the essential information needed for the feedback control system to function well is obtained not from a mathematical model, but through the input and output data of the plant in real-time [32]. In particular, the linear active disturbance rejection controller (LADRC), the linear form of ADRC, with a clear structure and sample parameter tuning method proposed by Gao [33], is gradually becoming a powerful competitor of the general industrial controller PID. At present, LADRC has been widely applied in servo-motor control [34], superheated steam temperature control of power plants [35], and multi-axis system control [36].

In this paper, an integrated control strategy is presented integrates model reference adaptive dead-zone inverse (MRADI) compensation and an error-based LADRC position synchronization regulator. Due to the capacity of AC to estimate unknown time-varying parameters, a model reference adaptive dead-zone inverse compensation control approach is introduced to promote each cylinder position’s tracking accuracy. Concurrently, an error-based LADRC is employed as the position synchronization error regulation to handle the motion coupling of the dual-cylinders. Intricate decoupling design is avoided to benefit from the total disturbance concept of LADRC. Furthermore, the error-based LADRC has a simple structure similar to PID and is particularly suitable for industrial practitioners.

The remaining content of this paper is organized as follows. In Section 2, the experimental platform of the dual-cylinders EHPS is established. Modeling and parameter identification are described. In Section 3, the model reference adaptive dead-zone inverse compensator is designed, and the convergence of the adaptive law is proven. In Section 4, the error-based LADRC position synchronization control regulator is developed. In Section 5, adequate comparative experiments are detailed, which were done to verify the effectiveness of the proposed control strategy. In Section 6, we summarize the paper and give conclusions.

## 2. System Description and Modeling

### 2.1. Experimental Platform Description

**Figure 1:**
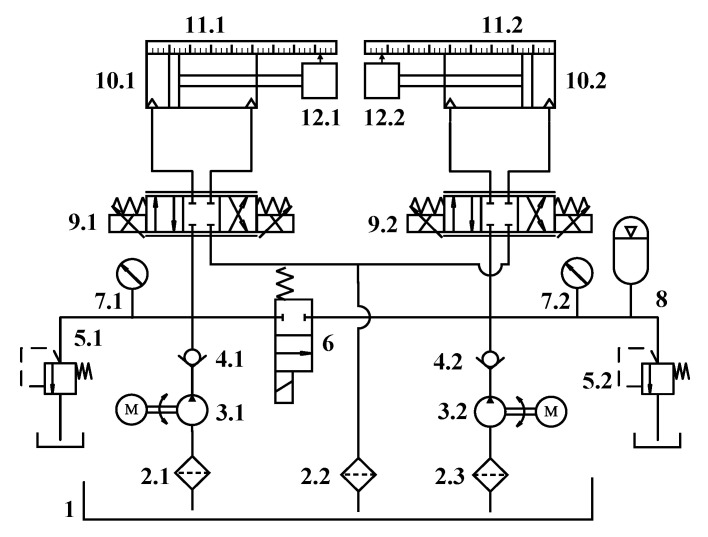
Hydraulic schematic of the experimental platform. (1) Tank. (2) Filter. (3) AC motor and pump. (4) Check valve. (5) Relief valve. (6) Two-position two-way directional valve. (7) Pressure gauge. (8) Accumulator. (9) Proportional directional valve. (10) Hydraulic cylinder. (11) Linear displacement sensor. (12) Inertia load.

The components and schematic of the investigated electro-hydraulic proportional position synchronization control system are illustrated in Figure 1. It consists of two symmetrical hydraulic systems. Still, each side of the hydraulic system contains an independent hydraulic power unit, an asymmetric hydraulic cylinder, an electro-hydraulic proportional direction valve, and a linear displacement sensor. Moreover, the hydraulic system also includes relief valves, check valves, oil filters, and accumulators, ensuring the system’s regular operation. The movement of each hydraulic cylinder is driven by an independent proportional valve. There are two control objectives: the first is that each cylinder accurately tracks the reference signal; the second is to minimize the synchronization error between the two cylinders.

### 2.2. Modeling

According to Figure 1, the left and the right valve-controlled hydraulic cylinder system structure is almost entirely consistent, so one of them was modeled. The EHPS mainly consists of five parts: a valve-controlled asymmetric hydraulic cylinder, a proportional directional valve, an amplifier, position feedback, and a dead-zone. Modeling was carried out with the following five aspects.

#### 2.2.1. Valve-Controlled Asymmetric Hydraulic Cylinder

The flow in both the positive and negative directions of the asymmetric hydraulic cylinder is discontinuous and needs to be considered separately. For the sake of simplicity, the following assumptions are proposed: (1) The proportional valve structure is symmetrical, and the valve orifice area gradient is equal; (2) the supply oil pressure is constant, and the return oil pressure is zero.

It is defined as the positive direction when the hydraulic cylinder piston rod is extended. Then the flow equations of the proportional valve in positive and negative directions can be described as follows [3]:

(1) $$\begin{array}{l} \left\{ \begin{array}{l} Q_{1}=C_{d}wx_{v}\sqrt{2(p_{s}-p_{1})/\rho }\\ Q_{2}=C_{d}wx_{v}\sqrt{2p_{2}/\rho } \end{array}\right.\;\dot{y}>0\\ \left\{ \begin{array}{l} Q_{1}=C_{d}wx_{v}\sqrt{2p_{1}/\rho }\\ Q_{2}=C_{d}wx_{v}\sqrt{2(p_{s}-p_{2})/\rho } \end{array}\right.\;\dot{y}<0 \end{array}$$

where $Q_{1}$ and $Q_{2}$ are the supplied flow of the rodless chamber and the return flow of the rod chamber, $C_{d}$ is the discharge coefficient of the proportional valve, $x_{v}$ is the valve spool displacement, $p_{s}$ is the supply oil pressure, *ρ* is the density of oil, $p_{1}$ is the rodless chamber pressure, and $p_{2}$ is the rod chamber pressure.

In the hydraulic cylinder motion process, the external leakage of oil is a trace amount, so it is ignored. The hydraulic cylinder flow continuity equations are given by [9]

(2) $$\left\{ \begin{array}{l} Q_{1}=A_{1}\dot{y}+C_{i}(p_{1}-p_{2})+(V_{01}+A_{1}y){\dot{p}}_{1}/\beta_{e}\\ Q_{2}=A_{2}\dot{y}+C_{i}(p_{1}-p_{2})-(V_{02}-A_{2}y){\dot{p}}_{2}/\beta_{e} \end{array}\right.$$

where y and $\dot{y}$ are the hydraulic cylinder position and velocity, $A_{1}$ and $A_{2}$ are the effective areas of the rodless chamber and the rod chamber, $C_{i}$ is the oil internal leakage coefficient, $\beta_{e}$ is the effective oil bulk modulus, and $V_{01}$ and $V_{02}$ are the initial volumes of two chambers.

The flow produced by leakage and the volume effect is much smaller than that produced by piston motion. From Equations (1) and (2), the flow ratio of two chambers n can be approximately written as

(3) $$\left\{ \begin{array}{l} \begin{array}{cc} n=Q_{2}/Q_{1}=A_{2}/A_{1}=\sqrt{p_{2}/\left(p_{s}-p_{1}\right)} & \dot{y}>0 \end{array}\\ \begin{array}{cc} n=Q_{2}/Q_{1}=A_{2}/A_{1}=\sqrt{\left(p_{s}-p_{2}\right)/p_{1}} & \dot{y}<0 \end{array} \end{array}\right.$$

Define $p_{L}=p_{1}-p_{2}$ is the load pressure, and combined with Equation (3) we can derive

(4) $$\begin{array}{l} \left\{ \begin{array}{l} p_{1}=\left(n^{2}p_{s}+p_{L}\right)/\left(1+n^{2}\right)\\ p_{2}=\left[n^{2}(p_{s}-p_{L})\right]/\left(1+n^{2}\right) \end{array}\right.\;\dot{y}>0\\ \left\{ \begin{array}{l} p_{1}=\left(p_{s}+p_{L}\right)/\left(1+n^{2}\right)\\ p_{2}=\left(p_{s}-p_{L}n^{2}\right)/\left(1+n^{2}\right) \end{array}\right.\;\;\;\dot{y}<0 \end{array}$$

Furthermore, the load flow is described as

(5) $$Q_{L}=\frac{Q_{1}+Q_{2}}{2}$$

From Equations (1) and (4), the uniform expression of $Q_{L}$ in the positive and negative directions is

(6) $$Q_{L}=aC_{d}wx_{v}\sqrt{\frac{1}{\rho }(p_{s}-\frac{x_{v}}{\left|x_{v}\right|}p_{L})}$$

where $a=\left(1+n\right)/\sqrt{2(1+n^{2})}$.

Linearize Equation (6); then $Q_{L}$ can be written as [7]

(7) $$Q_{L}=K_{q}x_{v}-K_{p}p_{L}$$

where $K_{q}$ is the flow gain coefficient and $K_{p}$ is the flow pressure coefficient. Their expressions are

(8) $$\left\{ \begin{array}{l} K_{q}=\frac{\partial Q_{L}}{\partial x_{v}}=aC_{d}w\sqrt{\left(p_{s}-p_{L0}\right)/\rho }\\ K_{p}=\frac{\partial Q_{L}}{\partial p_{L}}=\frac{aC_{d}wx_{v0}\sqrt{1/\rho }}{2\sqrt{p_{s}-p_{L0}}} \end{array}\right.$$

where $x_{v0}$ and $p_{L0}$ are the valve spool displacement and the load pressure in the operating point.

From Equations (2), (4) and (5), the flow continuity equation of the hydraulic cylinder can be expressed as [11]

(9) $$Q_{L}=A_{me}\dot{y}+C_{i}p_{L}+\frac{V_{e}}{4\beta_{e}}{\dot{p}}_{L}$$

where $A_{me}$ is the average piston area and $V_{e}$ is the equivalent volume of the hydraulic cylinder. Their expressions are

(10) $$A_{me}=\frac{(1+n)A_{1}}{2}$$

(11) $$V_{e}=A_{e}L=\frac{(1+n^{3})A_{1}L}{1+n^{2}}$$

where L is the stroke of hydraulic cylinder and $A_{e}$ is the equivalent piston area.

According to Newton’s second law, the hydraulic cylinder force balance equation is

(12) $$p_{1}A_{1}-p_{2}A_{2}=m\ddot{y}+b\dot{y}+Ky+F$$

where m is the equivalent mass, including the piston and the load; b denotes the viscous damping coefficient; K is the load stiffness coefficient; F is the unmodeled dynamics’ disturbance force, including nonlinear frictions and unknown external disturbances.

Substitute Equation (4) into Equation (12); then we obtain

(13) $$p_{L}=\frac{1}{A_{e}}(m\ddot{y}+b\dot{y}+Ky+f_{e})$$

where $f_{e}=F-f_{ad}$ is equivalent disturbance force; $f_{ad}$ is additional disturbance force, and its expression is

(14) $$f_{ad}=\left\{ \begin{array}{l} \frac{n^{2}(1-n)A_{1}}{1+n^{2}}p_{s}\;\dot{y}>0\\ \frac{(1-n)A_{1}}{1+n^{2}}p_{s}\;\;\;\;\dot{y}<0 \end{array}\right.$$

After the Laplace transform of the Equations (7), (9) and (13), the dynamic model of the valve-controlled asymmetric hydraulic cylinder can be described by

(15) $$Y(s)=\frac{K_{q}x_{v}-\frac{K_{\mathrm{ce}}}{A_{e}}(\frac{V_{e}}{4\beta_{e}K_{\mathrm{ce}}}s+1)f_{e}}{\frac{V_{e}m}{4\beta_{e}A_{e}}s^{3}+(\frac{V_{e}b}{4\beta_{e}A_{e}}+\frac{K_{\mathrm{ce}}m}{A_{e}})s^{2}+(\frac{V_{e}K}{4\beta_{e}A_{e}}+\frac{K_{ce}b}{A_{e}}+A_{me})s+\frac{K_{ce}K}{A_{e}}}$$

where $K_{\mathrm{ce}}=K_{p}+C_{i}$ is the total flow pressure coefficient and s is the Laplace operator.

Generally, the electro-hydraulic position control system has an inertial load, which means K=0. Additionally, the viscous damping coefficient b is minimal, so the inequality $K_{ce}b/A_{e}\ll A_{me}$ is satisfied. Therefore, Equation (15) can be simplified as [3]

(16) $$Y(s)=\frac{\frac{K_{q}}{A_{\mathrm{me}}}x_{v}-\frac{K_{\mathrm{ce}}}{A_{\mathrm{me}}A_{e}}(\frac{V_{e}}{4\beta_{e}K_{\mathrm{ce}}}s+1)f_{e}}{s(\frac{s^{2}}{\omega_{h}^{2}}+\frac{2\xi_{h}}{\omega_{h}}s+1)}$$

where $\omega_{h}=2\sqrt{A_{e}A_{\mathrm{me}}\beta_{e}/V_{e}m}$ is the natural frequency and $\xi_{h}=K_{\mathrm{ce}}\sqrt{m\beta_{e}/V_{e}A_{e}A_{\mathrm{me}}}+b\sqrt{V_{e}/m\beta_{e}A_{e}A_{\mathrm{me}}}/4$ is the damping ratio.

#### 2.2.2. Amplifier

The amplifier is responsible for converting input control voltage to current. The amplifier is regarded as a proportional part since the conversion speed is breakneck, and the equation can be given as

(17) $$i=K_{a}u$$

where i is the output current, $K_{a}$ is the amplification coefficient, and u is the input control voltage from the computer.

#### 2.2.3. Electro-Hydraulic Proportional Valve

In this paper, the proportional valve dead-zone is considered as a part of the whole system model (the benefits will be discussed later). The remaining proportional valve linear model can be simplified as a first-order inertial link [20], and it can be described as

(18) $$\frac{x_{v}}{i}=\frac{K_{v}}{Ts+1}$$

where $K_{v}$ is the gain coefficient and T is the time constant.

#### 2.2.4. Displacement Feedback

The linear displacement sensors are utilized to measure hydraulic cylinder displacement in this system. Due to the sensor’s sampling frequency being much higher than the closed-loop bandwidth of the control system, the sensor can be considered a proportional stage. It can be denoted as

(19) $$u_{s}=K_{s}y$$

where $u_{s}$ the linear displacement sensor feedback voltage and $K_{s}$ is the feedback gain coefficient.

#### 2.2.5. Dead-Zone Nonlinearity

**Figure 2:**
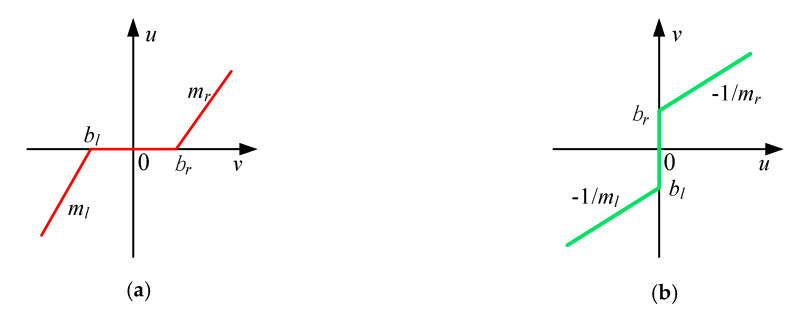
Dead-zone and dead-zone inverse: (**a**) dead-zone; (**b**) dead-zone inverse.

Generally, the electro-hydraulic proportional valve exists a spool overlap. When the spool displacement is less than the overlap, the flow is zero, resulting in the system output dead-zone phenomenon [21]. Therefore, the dead-zone effect of the proportional valve can be equivalent to the dead-zone between the system control input u and output y. The dead-zone is extracted from the linear model beneficial to the design of dead-zone parameters’ adaptive law. Accordingly, the dead-zone nonlinearity N(·) can be presented as [23]

(20) $$u=DZ(v)=\{\begin{array}{lll} m_{r}(v-b_{r}) & & v\geq b_{r}\\ 0 & & b_{l}<v<b_{r}\\ m_{l}(v-b_{l}) & & v\leq b_{l} \end{array}$$

where $m_{r}$ and $m_{l}$ are the right slope and left slope; $b_{r}$ and $b_{l}$ represent the right break-point and left break-point of the dead-zone, respectively. v is the input of the dead-zone. The dead-zone parameters are all unknown. In later, a dead-zone inverse $N^{-1}(\cdot )$ compensation will be designed to offset it. The graphical illustration of the dead-zone and dead-zone inverse is given in Figure 2.

**Figure 3:**
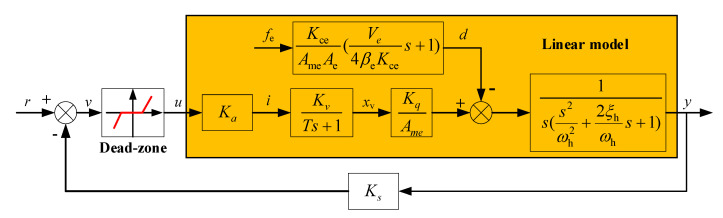
Block diagram of the electro-hydraulic proportional position control system.

According to the above models, each side of the electro-hydraulic proportional position control system model can be divided into the nonlinear dead-zone and the linear model. The block diagram is shown in Figure 3.

In Figure 3, it can be regarded that the electro-hydraulic proportional position synchronization control system consists of two subsystems represented as $G_{1}$ and $G_{2}$, respectively. The position synchronization error is defined as

(21) $$y_{e}=y_{1}-y_{2}$$

where $y_{1}$ and $y_{2}$ are left and right hydraulic cylinder position outputs.

Rewrite Equation (16) in a differential equation form, and combine it with Equation (21). Then the dual-hydraulic actuators dynamic model, including position synchronization error, can be described as

(22) $$\left\{ \begin{array}{l} y_{1}^{4}=a_{13}\dddot{y}+a_{12}\ddot{y}+a_{11}\dot{y}+a_{10}y+b_{1}u+d_{1}\\ y_{2}^{4}=a_{23}\dddot{y}+a_{22}\ddot{y}+a_{21}\dot{y}+a_{20}y+b_{2}u+d_{2}\\ y_{e}^{4}=y_{1}^{4}-y_{2}^{4} \end{array}\right.$$

where $a_{i3}=-(\frac{1}{T}+2\xi_{ih}\omega_{ih})$, $a_{i2}=-(\frac{2\omega_{ih}\xi_{ih}}{T}+\omega_{ih}^{2})$, $a_{i1}=-\frac{\omega_{ih}^{2}}{T}$, $a_{i0}=0$, $b_{i}=\frac{K_{q}K_{a}K_{v}\omega_{ih}^{2}}{TA_{me}}$, $d_{i}=\frac{K_{ce}\omega_{h}^{2}}{A_{me}A_{e}T}\left[\frac{V_{e}T}{4\beta_{e}K_{ce}}{\ddot{f}}_{e}+(\frac{V_{e}}{4\beta_{e}K_{ce}}+T){\dot{f}}_{e}+f_{e}\right]$, and i=1,2.

Ideally, the dynamic and static characteristics of the two subsystems should be consistent, since the structures of the systems $G_{1}$ and $G_{2}$ are entirely symmetrical. However, it backfires in the actual system due to the components’ installation positions, working conditions, wear, etc. Therefore, the position synchronization error is generated.

### 2.3. Identification of Model Parameters 

As is known to all, hydraulic system parameters exist with uncertainty, such as $C_{i}$, $\beta_{e}$, $K_{q}$, and b. Therefore, it is not easy to obtain precise model parameters. In the model reference adaptive dead-zone inverse compensation control algorithm, the plant model is demanded and obtained by the parameter identification method. In this paper, the forgetting factor recursive least square approach (FFRLS), which can avoid “data saturation” in the parameter estimation process, is applied to estimates the critical parameters of the system. For simplicity of analysis, Assuming that $f_{e}=0$, the system model is reduced to a fourth-order transfer function, and it satisfies the extended auto-regressive model (ARX).

(23) $$G(s)=\frac{Y(s)}{U(s)}=\frac{K_{q}K_{a}K_{v}/A_{me}}{s(Ts+1)(\frac{s^{2}}{\omega_{h}^{2}}+\frac{2\xi_{h}}{\omega_{h}}s+1)}$$

The discrete difference form of Equation (23) can be obtained via the delay theorem of z transformation and rewritten as the least-squares format

(24) $$y(k)=H^{T}(k)\theta$$

where u(k) and y(k) are the inputs and outputs of the system at different times, respectively. $H^{T}(k)$ and θ can be noted as

(25) $$\left\{ \begin{array}{l} H^{T}(k)=[-y(k-1),-y(k-2),-y(k-3),-y(k-4),\\ u(k-1),u(k-2),u(k-3),u(k-4)]\\ \theta =\left[\begin{array}{llllllll} a_{1} & a_{2} & a_{3} & a_{4} & b_{1} & b_{2} & b_{3} & b_{4} \end{array}\right] \end{array}\right.$$

Parameter identification for the EHPS can be regarded as the identification of parameters θ. Selecting the optimal estimation performance criterion function J to be

(26) $$J=J(\theta )=\sum_{k=1}^{N}\lambda^{N-k}{\left[y(k)-H^{T}(k)\hat{\theta }\right]}^{2}$$

where λ is the forgetting factor, and 0<λ≤1, $\hat{\theta }=\left[\begin{array}{llllllll} {\hat{a}}_{1} & {\hat{a}}_{2} & {\hat{a}}_{3} & {\hat{a}}_{4} & {\hat{b}}_{1} & {\hat{b}}_{2} & {\hat{b}}_{3} & {\hat{b}}_{4} \end{array}\right]$ is the estimate of θ.

The FFRLS expression is shown as

(27) $$\left\{ \begin{array}{l} \hat{\theta }(k)=\hat{\theta }(k-1)+K(k)[y(k)-H^{T}(k)\hat{\theta }(k-1)]\\ K(k)=P(k-1)H(k)[H^{T}(k)P(k-1)H(k)+\lambda {]}^{-1}\\ P(k)=\lambda^{-1}[I-K(k)h^{T}(k)]P(k-1) \end{array}\right.$$

**Figure 4:**
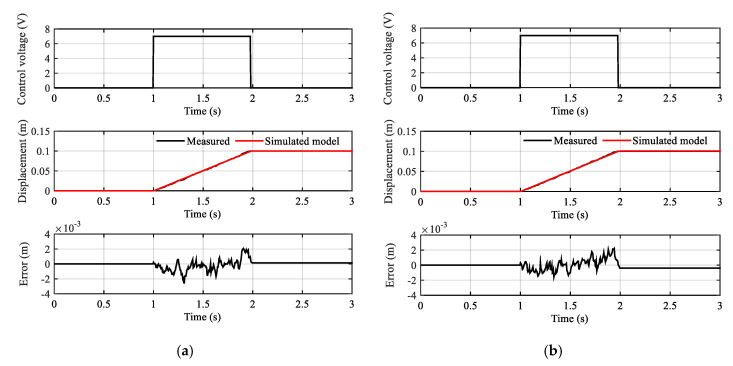
The fitting results: (**a**) Control input signal and fitting curves of the subsystem $G_{1}$; (**b**) Control input signal and fitting curves of the subsystem $G_{2}$.

In the identification process, the step control signal of 7 V is employed. The parameter identification results are shown in Table 1. The input control signal and fitting results are displayed in Figure 4.

See in Figure 4 the identification result curves of the subsystems $G_{1}$ and $G_{2}$ indicating that the measured displacements and simulation of the estimation model have a high coincidence, and the error of the displacement almost does not exceed ±4 mm. The fit to estimation data was 98.9% and 98.84%, respectively. Therefore, it can be inferred that the identification model is credible for this system.

## 3. Model Reference Adaptive Dead-Zone Inverse Controller

### 3.1. Design of Controller

**Figure 5:**
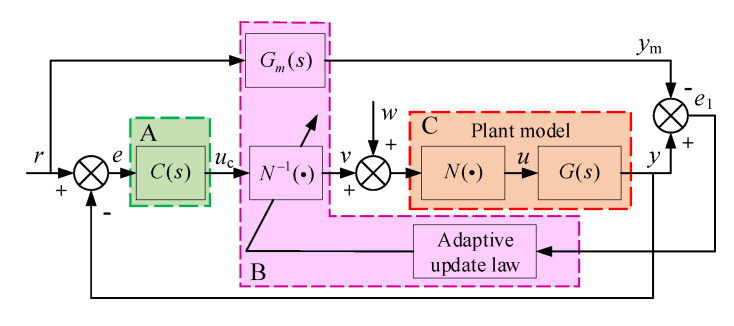
Control block diagram of model reference adaptive dead-zone inverse control.

In this section, a model reference adaptive dead-zone inverse controller (MRADIC) is designed for each channel. In Figure 5, the control structure consists of three parts: (A) a position controller, (B) a model reference adaptive dead-zone inverse controller, and (C) a plant model. In Figure 5, the r is the expected trajectory signal from trajectory generator; C(s) is the position controller, and its output is $u_{c}$; $N^{-1}(\cdot )$ is dead-zone inverse, and its output is v; $G_{m}(s)$ is the reference model; the plant includes the linear part G(s) and dead-zone N(·); e is the error between position output y and expected signal r; u is the input control signal of G(s); $e_{1}$ is the error between G(s) and $G_{m}(s)$. In addition, the dead-zone inverse parameters’ adaptive law is also involved.

Firstly, a PI controller is utilized as the position controller for the EHPS because of PID’s simple linear control structure and excellent industrial applicability. The structure of the PI controller can be denoted as

(28) $$C(s)=k_{p}+k_{i}\frac{1}{s}$$

According to Equation (20), the dead-zone inverse can be expressed as

(29) $$v=N^{-1}(u_{c})=\left\{ \begin{array}{ll} \frac{u_{c}+{\hat{m}}_{r}{\hat{b}}_{r}}{{\hat{m}}_{r}} & u_{c}>0\\ 0 & u_{c}=0\\ \frac{u_{c}+{\hat{m}}_{l}{\hat{b}}_{l}}{{\hat{m}}_{l}} & u_{c}<0 \end{array}\right.$$

where v is the dead-zone inverse output, $u_{c}$ is the controller C(s) output, and ${\hat{m}}_{r}$, ${\hat{b}}_{r}$, ${\hat{m}}_{l}$, and ${\hat{b}}_{l}$ are the estimates of dead-zone parameters.

Since the parameters of the dead-zones are unknown and are affected by hydraulic components’ abrasion, the working pressure, and the running status during the operation of the system, the dead-zone settings change, resulting in compensation deviations. To solve this problem, an adaptive law was designed to update the parameters in real-time.

Define the unknown dead-zone parameters vector as $\delta ={\left[\delta_{1},\delta_{2},\delta_{3},\delta_{4}\right]}^{T}={\left[m_{r},m_{r}b_{r},m_{l},m_{l}b_{l}\right]}^{T}$. According to Figure 5, the dead-zone characteristic can be linearly parameterized as

(30) $$u(t)=N(v(t))=-\delta^{T}(t)\omega (t)$$

where $\omega (t)={\left[-\chi (t)v(t),\chi (t),(\chi (t)-1)v(t),1-\chi (t)\right]}^{T}$, in which the indicator function χ(t) is defined as

(31) $$\chi (t)=\left\{ \begin{array}{c} 1,\;\;u_{c}(t)\geq 0\\ 0,\;\;u_{c}(t)<0 \end{array}\right.$$

Since the parameters δ are unknown, define $\hat{\delta }={\left[{\hat{\delta }}_{1},{\hat{\delta }}_{2},{\hat{\delta }}_{3},{\hat{\delta }}_{4}\right]}^{T}={\left[{\hat{m}}_{r},{\hat{m}}_{r}{\hat{b}}_{r},{\hat{m}}_{l},{\hat{m}}_{l}{\hat{b}}_{l}\right]}^{T}$ as the estimate of δ. Hence, the actual control input $u_{c}(t)$ to the plant can be described as

(32) $$u_{c}(t)=-{\hat{\delta }}^{T}(t)\omega (t)$$

Therefore, the dead-zone compensation error between u and $u_{c}$ is

(33) $$\Delta u(t)=u(t)-u_{c}(t)={\left(\hat{\delta }-\delta \right)}^{T}\hat{\omega }(t)+d_{N}(t)$$

where $d_{N}(t)=m_{r}s_{r}(t)(v(t)-b_{r})+m_{l}s_{l}(v(t)-b_{l})$ is the unmodeled deviation, and

(34) $$\begin{array}{l} s_{r}(t)=\left\{ \begin{array}{l} -1,\;\;0\leq v(t)<b_{r}\\ 0. \end{array}\right.\\ s_{l}(t)=\left\{ \begin{array}{l} -1,\;\;b_{l}<v(t)<0\\ 0. \end{array}\right. \end{array}$$

According to the Equations (32)–(34), $d_{N}(t)$ is bounded and satisfied [23]:

(1) When ${\hat{b}}_{r}>b_{r}$ or ${\hat{b}}_{l}>b_{l}$,$d_{N}(t)=0$;

(2) When $v(t)\geq b_{r}$ or $v(t)\leq b_{l}$, $d_{N}(t)=0$;

(3) $d_{N}(t)$ approaches zero, depending on the dead-zone estimate parameter $\hat{\delta }$. 

The output feedback is applied to design the adaptive updating law for $\hat{\delta }$. Ideally, the parameters of dead-zone inverse estimation are equal to the actual values. Then we obtained the following result.

(35) $$\left\{ \begin{array}{l} \Delta u(t)=d_{N}(t)=0\\ u(t)=u_{c}(t) \end{array}\right.$$

According to the feedback transformation, the reference model in Figure 5 can be derived.

(36) $$G_{m}(s)=\frac{C(s)G(s)}{1+C(s)G(s)}$$

The error caused by dead-zone inverse incomplete compensation is defined as [25]

(37) $$e_{1}(t)=H(s)[{\left(\hat{\delta }-\delta \right)}^{T}\omega +d_{N}](t)$$

where $H(s)=\frac{G(s)}{1+C(s)G(s)}$.

In [23,25], the gradient projection adaptive law has been designed to update the adaptive inverse dead-zone parameters, and it can be represented as

(38) $$\dot{\hat{\delta }}(t)=-\frac{\Gamma \zeta (t)\varepsilon (t)}{1+\zeta^{T}(t)\zeta (t)+\xi^{2}(t)}-\Gamma \sigma (\hat{\delta }(t))\hat{\delta }(t)$$

where Γ is a positive definite matrix, and $\Gamma^{T}=\Gamma >0$.

(39) $$\sigma (\hat{\delta }(t))=\left\{ \begin{array}{ll} 0 & \left\|\hat{\delta }(t)\right\|<M_{0}\\ \sigma_{0}\left(\frac{\left\|\hat{\delta }(t)\right\|}{M_{0}}-1\right) & M_{0}\leq \left\|\hat{\delta }(t)\right\|<2M_{0}\\ \sigma_{0} & \left\|\hat{\delta }(t)\right\|\geq 2M_{0} \end{array}\right.$$

(40) $$\varepsilon (t)=e_{1}(t)+\xi (t)$$

(41) $$\xi (t)={\hat{\delta }}^{T}(t)\zeta (t)-H(s)[{\hat{\delta }}^{T}\omega ](t)$$

(42) ζ(t)=H(s)ω(t)

In Equation (39), the parameters $\sigma_{0}$ and $M_{0}$ need to be designed. Note that the parameter $\sigma_{0}>0$, and the parameter $M_{0}$ is the upper bound of the dead-zone parameters’ Euclidean norm ‖δ(t)‖, which is determined by a priori knowledge.

### 3.2. Convergence Analysis of Adaptive Law

The dead-zone inverse parameters adaptive law can ensure the convergence of parameter estimation errors, and the convergence is proved by the Lyapunov method. The parameters estimation errors are defined as:

(43) $$E(t)=\hat{\delta }(t)-\delta (t)$$

By substituting (37) into (40), ε(t) is written as:

(44) $$\begin{array}{cc} \varepsilon (t) & =H(s)[(\hat{\delta }-\delta {)}^{T}\omega +d_{N}](t)+{\hat{\delta }}^{T}(t)\zeta (t)-H(s)[{\hat{\delta }}^{T}\omega ](t)\\ & =H(s){\hat{\delta }}^{T}(t)\omega (t)-H(s){\hat{\delta }}^{T}(t)\omega (t)+H(s)d_{N}(t)+\;{\hat{\delta }}^{T}(t)H(s)\omega (t)-H(s)[{\hat{\delta }}^{T}\omega ](t)\\ & ={\hat{\delta }}^{T}(t)H(s)\omega (t)-H(s){\hat{\delta }}^{T}(t)\omega (t)+H(s)d_{N}(t)\\ & =({\hat{\delta }}^{T}(t)-{\hat{\delta }}^{T}(t))H(s)\omega (t)+H(s)d_{N}(t)\\ & =E^{T}(t)\zeta (t)+d_{1}(t) \end{array}$$

where $d_{1}(t)=H(s)d_{N}(t)$.

Select the Lyapunov function as

(45) $$V(E(t))=\frac{1}{2}E^{T}(t)\Gamma^{-1}E(t)$$

By taking the derivatives of both sides, we obtain

(46) $$\begin{array}{cc} \dot{V}(E(t)) & =\frac{1}{2}{\dot{E}}^{T}(t)\Gamma^{-1}E(t)+\frac{1}{2}E^{T}(t)\Gamma^{-1}\dot{E}(t)\\ & =E^{T}(t)\Gamma^{-1}\dot{E}(t)\\ & =-\frac{E^{T}(t)\xi (t)\varepsilon (t)}{1+\zeta^{T}(t)\zeta (t)+\xi^{2}(t)}-f(\hat{\delta }(t))E^{T}(t)\hat{\delta }(t) \end{array}$$

Since $-{\left(\varepsilon (t)/\sqrt{2}-d_{1}(t)/\sqrt{2}\right)}^{2}\leq 0$, then

(47) $$-\varepsilon^{2}(t)+d_{1}(t)\varepsilon (t)\leq -\frac{\varepsilon^{2}(t)}{2}+\frac{d_{1}^{2}(t)}{2}$$

Combining (44) and (47), we obtain

(48) $$\dot{V}(\varphi (t))\leq -\frac{\varepsilon^{2}(t)-d_{1}^{2}(t)}{2(1+\zeta^{T}(t)\zeta (t)+\xi^{2}(t))}-f(\hat{\delta }(t))E^{T}(t)\hat{\delta }(t)$$

There are two cases: 

(1) When $\left\|\hat{\delta }(t)\right\|>M_{0}$, $f(\hat{\delta }(t))E^{T}(t)\hat{\delta }(t)>0$;

(2) When $\left\|\hat{\delta }(t)\right\|\leq M_{0}$, $f(\hat{\delta }(t))E^{T}(t)\hat{\delta }(t)=0$.

Therefore, existing $V_{0}>0$, when $V>V_{0}$, the Lyapunov function˙$\dot{V}<0$. According to the Lyapunov stability theory, the parameter estimates of dead-zone inverse converges to the actual values.

## 4. LADRC Synchronization Controller

### Controller Design

**Figure 6:**
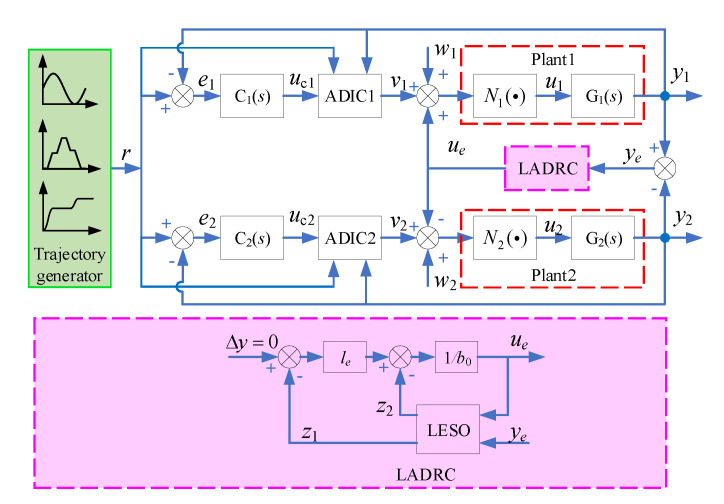
Structure block diagram of the position synchronization control system.

For the position synchronization problem of dual-hydraulic actuators, a synchronization controller is introduced. The synchronization error-based LADRC is designed to generate the synchronous control signal $u_{e}$, which can compensate for the position synchronization error. The integrated control structure is shown in Figure 6.

For the electro-hydraulic position servo system with an inertial load, the hydraulic cylinder position can be calculated using flow rather than pressure [37]. Besides, we are more concerned with the steady-state accuracy of synchronous motion rather than the system’s higher-order dynamics. Then, combining to Figure 6, the dynamic model of the system, describing in Equation (22), can be rewritten as

(49) $$\left\{ \begin{array}{l} {\dot{y}}_{1}=-\frac{1}{a_{11}}[-{\ddddot{y}}_{1}+a_{13}{\dddot{y}}_{1}+a_{12}{\ddot{y}}_{1}+\omega_{1}+d_{1}+b_{1}(N_{1}(v_{1})+u_{e})]\\ {\dot{y}}_{2}=-\frac{1}{a_{21}}[-{\ddddot{y}}_{2}+a_{23}{\dddot{y}}_{2}+a_{22}{\ddot{y}}_{2}+\omega_{2}+d_{2}+b_{2}(N_{2}(v_{2})-u_{e})]\\ {\dot{y}}_{e}={\dot{y}}_{1}-{\dot{y}}_{2} \end{array}\right.$$

where $w_{1}$ and $w_{2}$ express the unknown external disturbances of the subsystem $G_{1}$ and $G_{2}$, respectively.

In the ADRC framework, the total disturbances of system $G_{1}$ and system $G_{2}$ can be defined as

(50) $$\left\{ \begin{array}{l} {\dot{y}}_{1}=\underset{f_{1}}{\underbrace{-\frac{1}{a_{11}}[-{\ddddot{y}}_{1}+a_{13}{\dddot{y}}_{1}+a_{12}{\ddot{y}}_{1}+\omega_{1}+d_{1}+b_{1}N_{1}(v_{1})]}}-\frac{b_{1}}{a_{11}}u_{e}\\ {\dot{y}}_{2}=\underset{f_{2}}{\underbrace{-\frac{1}{a_{21}}[-{\ddddot{y}}_{2}+a_{23}{\dddot{y}}_{2}+a_{22}{\ddot{y}}_{2}+\omega_{2}+d_{2}+b_{2}N_{2}(v_{2})]}}+\frac{b_{2}}{a_{21}}u_{e} \end{array}\right.$$

Then, the position synchronization system model is transformed into

(51) $$\left\{ \begin{array}{l} {\dot{y}}_{1}=f_{1}({\ddddot{y}}_{1},{\dddot{y}}_{1},{\ddot{y}}_{1},\omega_{1},d_{1},N_{1}(v_{1}))+{\bar{b}}_{1}u_{e}\\ {\dot{y}}_{2}=f_{2}({\ddddot{y}}_{2},{\dddot{y}}_{2},{\ddot{y}}_{2},\omega_{2},d_{2},N_{2}(v_{2}))+{\bar{b}}_{2}u_{e}\\ {\dot{y}}_{e}=f_{e}(f_{1},f_{2})+({\bar{b}}_{1}-{\bar{b}}_{2})u_{e} \end{array}\right.$$

where $f_{e}(f_{1},f_{2})$ is a nonlinear synthesis function of the internal unmodeled dynamics and unknown external disturbances for the position synchronization system. Accordingly, $f_{1}({\ddddot{y}}_{1},{\dddot{y}}_{1},{\ddot{y}}_{1},\omega_{1},d_{1},N_{1}(v_{1}))$ and $f_{2}({\ddddot{y}}_{2},{\dddot{y}}_{2},{\ddot{y}}_{2},\omega_{2},d_{2},N_{2}(v_{2}))$ are the nonlinear synthesis functions of the subsystem $G_{1}$ and $G_{2}$. ${\bar{b}}_{1}=-b_{1}/a_{11}$ and ${\bar{b}}_{2}=b_{2}/a_{21}$.

After such processing, the motion coupling existing between the two cylinders is considered as a lot to handle and simplified the complex control problem. That is the advantage of ADRC in dealing with system model imprecision, uncertainty, and unknown external disturbances.

The total disturbance of the synchronization system is estimated by the linear extended state observer (LESO) [33]. Considering the estimated deviation of the control gain $\left({\bar{b}}_{1}-{\bar{b}}_{2}\right)$, the position synchronization error can be rewritten as

(52) $${\dot{y}}_{e}={\dot{y}}_{1}-{\dot{y}}_{2}=\underset{f^{*}}{\underbrace{f_{e}(\cdot )+({\bar{b}}_{1}-{\bar{b}}_{2}-b_{0})u_{e}}}+b_{0}u_{e}$$

where $f^{*}$ is the total disturbance of the position synchronization system, $b_{0}$ is the estimate of $\left({\bar{b}}_{1}-{\bar{b}}_{2}\right)$.

It is clear that $f^{*}$ is differentiable and bounded in the actual system. Define $x_{1}=y_{e}$ and $x_{2}=f^{*}$; then the state equation of Equation (52) can be described as:

(53) $$\left\{ \begin{array}{l} {\dot{x}}_{1}=x_{2}+b_{0}u_{e}\\ {\dot{x}}_{2}=h \end{array}\right.$$

where $x_{1}$ and $x_{2}$ are the state variables, and $h={\dot{f}}^{*}$. 

A LESO is designed for the system described in Equation (53)

(54) $$\left\{ \begin{array}{l} {\dot{z}}_{1}=z_{2}-\beta_{1}(z_{1}-y_{e})+b_{0}u_{e}\\ {\dot{z}}_{2}=-\beta_{2}(z_{1}-y_{e}) \end{array}\right.$$

where $z_{1}$ and $z_{2}$ are estimates of the state variables $x_{1}$ and $x_{2}$, and $\beta_{1}$ and $\beta_{2}$ are the observer gains. Generally, $\left[\beta_{1},\beta_{2}]=[2\omega_{o},\omega_{o}^{2}\right]$, in which $\omega_{o}$ is the observer bandwidth [33]. It can realize $z_{1}\to y_{e}$ and $z_{2}\to f^{*}$ through tuning the parameter $\omega_{o}$.

Define $e_{i}=x_{i}-z_{i},i=1,2$ as the state estimation errors, and the error equation can be derived.

(55) $$\dot{\eta }=\omega_{o}A\eta +\frac{B}{\omega_{o}^{2}}h$$

where $\eta ={\left[\eta_{1},\eta_{2}\right]}^{T}={\left[e_{1},e_{2}/\omega_{o}\right]}^{T}$, $B={\left[0,1\right]}^{T}$, and $A=\left[\begin{array}{cc} -2 & 0\\ -1 & 0 \end{array}\right]$.

Since the matrix A is Hurwitz, there exists a positive definite symmetric matrix P that satisfies the Lyapunov equation $A^{T}P+PA=-I$, in which I is the identity matrix. According to the existing theoretical analysis results in [9], it can be inferred that the LESO in Equation (54) is stable. The estimation errors can be made arbitrarily small by increasing the observer bandwidth $\omega_{o}$.

Then, the position synchronization control signal $u_{e}$ is designed as

(56) $$u_{e}=\left(u_{0}-z_{2}\right)/b_{0}$$

where $u_{0}$ is the control law output signal. 

Ideally, the total disturbance $f^{*}$ can be accurately estimated by the LESO output signal $z_{2}$. Then, substituting Equation (56) into Equation (52), we obtain ${\dot{y}}_{e}=(f^{*}-z_{2})+u_{0}\approx u_{0}$. Now, the position synchronization system is simplified as a single integrator structure that is easy to be controlled. Generally, the proportional controller is adopted. The reference input signal is set to zero since the control target of position synchronization is $y_{e}\to 0$. The following equation can be derived.

(57) $$u_{0}=l_{e}(0-z_{1})$$

where $l_{e}$ is the only parameter that needed to be designed.

## 5. Experiment

### 5.1. Experimental Setup

**Figure 7:**
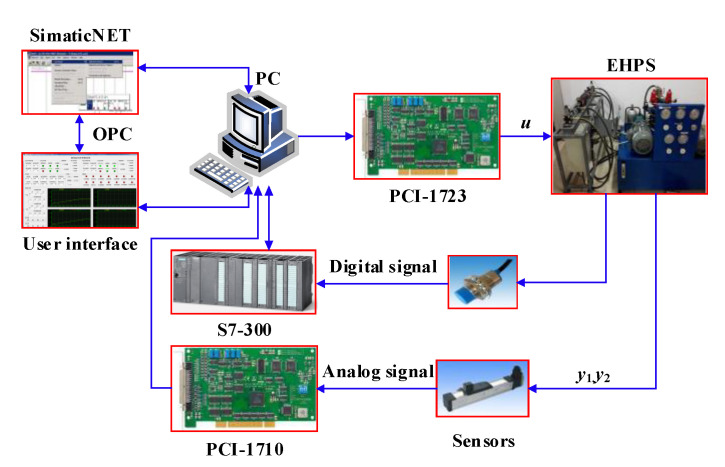
The experimental platform of electro-hydraulic synchronous control system.

In order to further verify the effectiveness and practicability of the proposed method in engineering applications, sufficient contrast experiments were performed on the electro-hydraulic proportional position synchronous control experimental platform. The physical diagram of the experimental platform is shown in Figure 7, namely, the electric control system and the hydraulic driving system.

The hydraulic system has been introduced in detail in Section 2. The electrical control system adopts the hardware structure of “industrial PC (IPC) + PLC.” The two parts are physically independent of each other but data interaction takes place through software. The PLC control system was employed to perform process control and collect digital signals. It is comprised of Siemens S7-300 CPU, two Siemens S7-321 digital signal input modules, and two Siemens S7-322 digital signal output modules. The IPC is responsible for real-time closed-loop control and acquisition of analog signals. Simultaneously, the Advantech data acquisition (DAQ) control modules PCI-1710 and PCI-1723 are embedded in IPC. The PCI-1710 card is a 12-bit analog signal acquisition module that meets the demand for real-time detection of displacement and pressure signal of the hydraulic drive system. The PCI-1723 is a 16-bit analog output card. It is responsible for the output voltage signal of –10 to 10 V to the proportional reversing valve.

In terms of software, the user interface based on VC++ obtains process signals from PLC through object linking and embedding for process control (OPC) technology. OPC technology establishes a data access interface standard with nothing to do with hardware and driver for communication between different industrial control system software. The server/client operating mode was adopted. In this design, the self-designed application is the client access SIMATIC NET, which is the OPC server storing data from PLC. In this way, the data of PLC and IPC are unified. The real-time performance of position closed-loop control can be ensured through such a software structure because of the high-speed data transfer of DAQ modules. The closed-loop real-time control period was 5 ms. The specifications of the electrical and hydraulic hardware components are provided in Table 2.

### 5.2. Control Effect of the Dead-Zone Adaptive Inverse Algorithm

**Figure 8:**
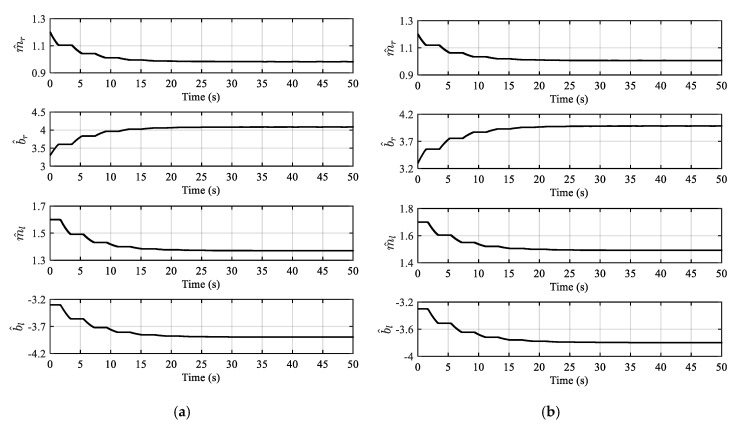
The convergence results of dead-zone parameters: (**a**) Dead-zone parameter estimation curve of hydraulic actuator 1; (**b**) dead-zone parameter estimation curve of hydraulic actuator 2.

Firstly, the effectiveness of the model reference dead-zone adaptive inverse control algorithm is verified. The sinusoidal desired trajectory r=0.05sin(0.25πt) mm is adopted. The convergence results of dead-zone parameters are shown in Figure 8. It is not difficult to find that all the dead-zone parameters are bounded and converged to a certain fixed value. However, the parameter convergence process is stepped, which is designed to avoid frequent reversing of the proportional valve.

**Figure 9:**
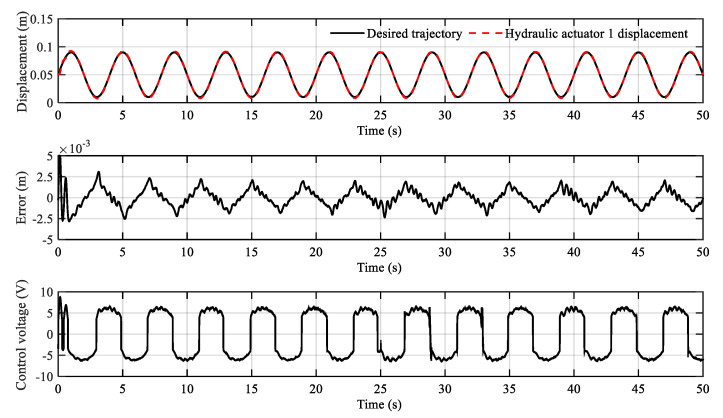
Sinusoidal trajectory tracking curve of hydraulic actuator 1.

**Figure 10:**
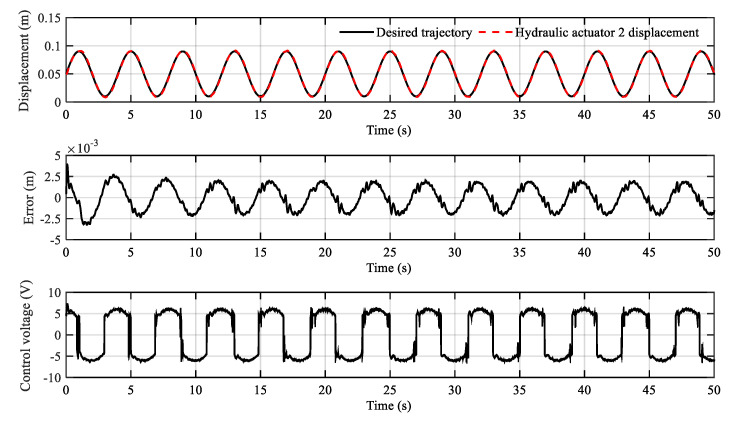
Sinusoidal trajectory tracking curve of hydraulic actuator 2.

The tracking results of the sinusoidal trajectory are shown in Figure 9 and Figure 10. It can be found that the tracking error decreases gradually over time. The tracking error remains within the range of ±2.5 mm after about 15 s. In addition, due to the discontinuity of the dead-zones, the control voltage curve will vibrate when the cylinder piston rod moves in the opposite direction. In general, the sinusoidal tracking motion, which is difficult for a proportional control system, is realized by dead-zone compensation.

### 5.3. The Synchronous Control Performance of LADRC

The synchronous control performance of the LADRC synchronization controller (LADRC-SC) was compared with the following two controllers, respectively: the without synchronization controller (W-SC) and PID synchronization controller (PID-SC). In the experiment, the stairstep signal with different slope and step signals, which are prevalent motion form in the industrial applications, was selected as the desired trajectory.

Additionally, there were three statistical indicators, which were employed to analyze the control performance of the proposed control approach, i.e., the maximum absolute value $M_{e}$, average value $\mu_{e}$, and standard deviation $\sigma_{e}$ of the synchronization errors. The detailed introductions are listed respectively as [26]:

(1) The maximal absolute value is utilized as an index of evaluation for the tracking error and is described as

(58) $$M_{e}=\underset{i=1,\ldots ,N}{\max}\{\left|e(i)\right|\}$$

where N represents the number of the recorded signals.

(2) The average tracking error can be described as

(59) $$\mu_{e}=\frac{1}{N}\sum_{i=1}^{N}\left|e(i)\right|$$

it is used to measure the average tracking performance of the tested controller.

(3) The standard deviation of the tracking error can be defined as

(60) $$\sigma_{e}=\sqrt{\frac{1}{N}\sum_{i=1}^{N}{\left(\left|e(i)\right|-\mu_{e}\right)}^{2}}$$

it is employed to measure the deviation level of the tracking errors.

In the designed control scheme, a PI controller is selected as the position controller. For simplicity, the PI controllers’ parameters are uniformly tuned as $k_{p}=900.0$ and $k_{i}=20.0$ thanks to regular experience. The initial parameters of dead-zone of the dual-hydraulic actuators were respectively selected as $\delta_{10}={\left[\begin{array}{cccc} 1.2 & 3.96 & 1.6 & -5.28 \end{array}\right]}^{T}$ and $\delta_{20}={\left[\begin{array}{cccc} 1.2 & 3.96 & 1.7 & -5.61 \end{array}\right]}^{T}$. The parameters of dead-zone adaptive law were uniformly selected as $\sigma_{0}=50$ $M_{0}=10$ and $\Gamma =60I_{4}$. Besides, The LADRC-SC parameters were selected as $l_{e}=20.0$, $\omega_{o}=20$, and $b_{0}=1.5$. The PID-SC parameters were selected as $k_{ps}=80.0$ and $k_{is}=5.0$.

In order to avoid overshoot caused by an excessive initial error when choosing the step signal, the transition process is arranged through the differential tracking (TD), and the specific expression is as follows [30]

(61) $$\left\{ \begin{array}{l} {\dot{v}}_{1}=v_{2}\\ {\dot{v}}_{2}=\mathrm{fhan}\left(v_{1}-r,v_{2},r_{0},h_{0}\right) \end{array}\right.$$

where $v_{1}$ is the tracking of the input signal r, $v_{2}$ is the differential of the input signal, r is the expect tracking signal, and the function $\mathrm{fhan}\left(x_{1},x_{2},r_{0},h_{0}\right)$ can be expressed as

(62) $$\left\{ \begin{array}{l} d=r_{0}h_{0}^{2}\\ a_{0}=h_{0}x_{2}\\ y=x_{1}+a_{0}\\ a_{1}=\sqrt{d(d+8\left|y\right|)}\\ a_{2}=a_{0}+\mathrm{sign}(y)(a_{1}-d)/2\\ s_{y}=[\mathrm{sign}(y+d)-\mathrm{sign}(y-d)]/2\\ a=(a_{0}+y-a_{2})s_{y}+a_{2}\\ s_{a}=[\mathrm{sign}(a+d)-\mathrm{sign}(a-d)]/2\\ \mathrm{fhan}=-x_{d}[a/d-sign(a)]s_{a}-r_{0}sign(a) \end{array}\right.$$

where $x_{1},x_{2}$ are the state variables of the function fhan, $r_{0},h_{0}$ are the parameters of the function fhan, and sign(x) is the symbolic function as

(63) $$\mathrm{sign}(x)=\left\{ \begin{array}{cc} 1 & x>0\\ 0 & x=0\\ -1 & x<0 \end{array}\right.$$

**Figure 11:**
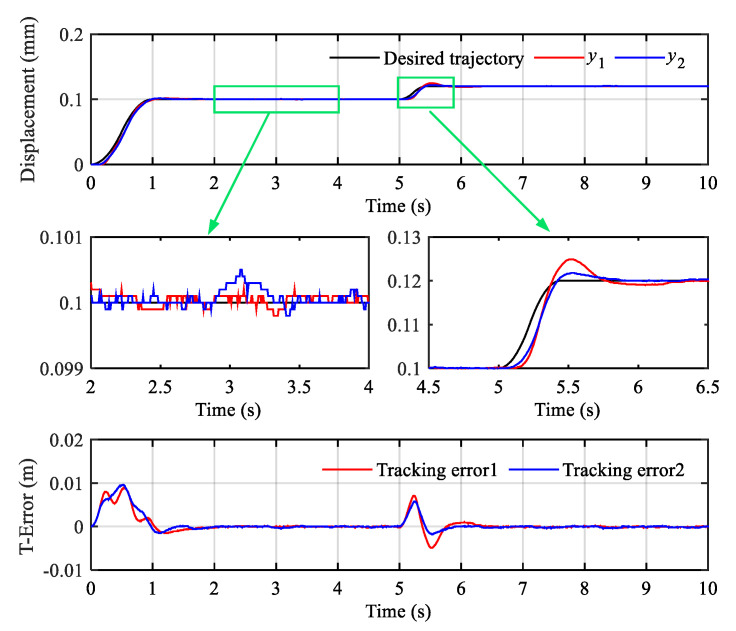
Position synchronization control results of the step signal.

The smoothed desired trajectory started at 1 s and jumped from 0.1 m to 0.12 m at 6 s. The response curve and tracking error (T-Error) curve are shown in Figure 11. 

**Figure 12:**
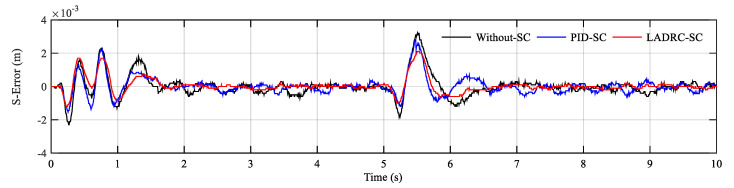
Synchronization error comparison results of the step signal.

In Figure 11, $y_{1}$ and $y_{2}$ represent the displacement output of hydraulic actuator 1 and hydraulic actuator 2, respectively. In order to display the details of the tracking curves clearly, local magnification of steady and transient processes is given. It can be found that the hydraulic cylinder actuators can quickly reach the target position with small overshoot and maintain low position steady-state error. Simultaneously, the synchronization error (S-Error) curve comparison results of three controllers are demonstrated in Figure 12. It can be seen that LADRC-SC has the minimum synchronization error, followed by the PID-SC.

**Figure 13:**
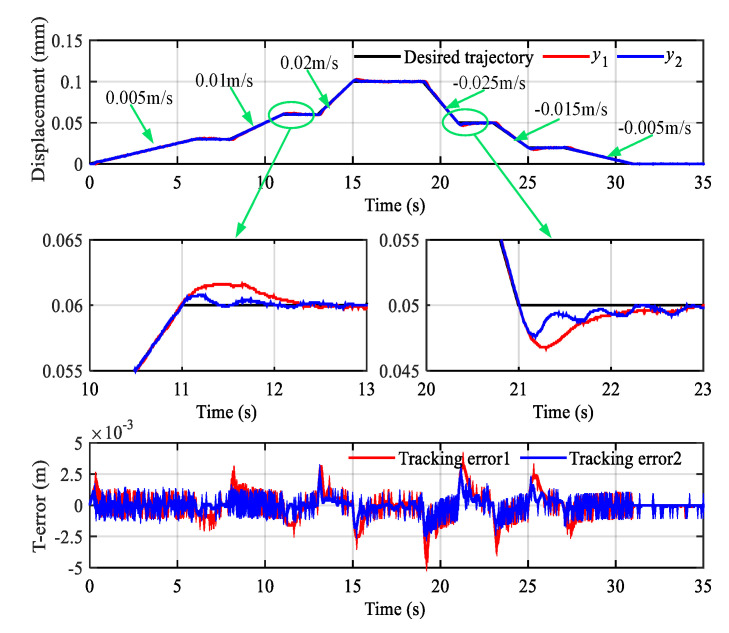
Position synchronization control results of the slope signal.

Continuous slope signals with different slopes verified the performance of low-speed synchronous control. The results are shown in Figure 13, in which $y_{1}$ and $y_{2}$ still represent the displacement output of hydraulic actuator 1 and hydraulic actuator 2, respectively. When the piston of the hydraulic cylinder is extended, the slope is selected as 0.005 m/s, 0.01 m/s, and 0.015 m/s. Accordingly, when the cylinder piston retracts, the slope is selected as −0.025 m/s, −0.015 m/s, and −0.005 m/s. When the slope changes during the movement, the overshoot of position tracking lead to a larger error, but it can remain within ±2.5 mm.

**Figure 14:**
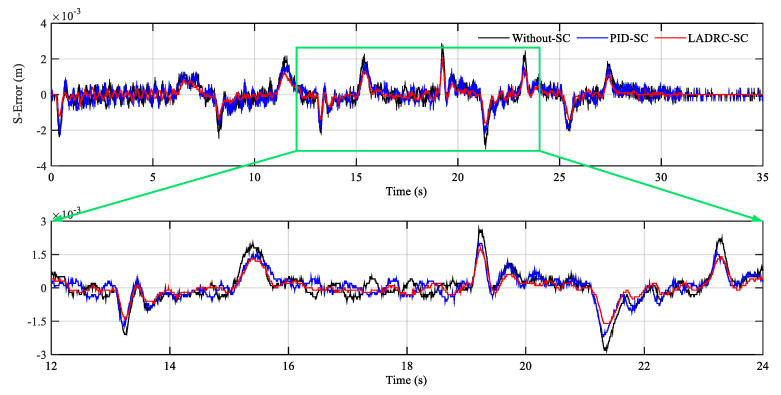
Synchronization error comparison results of the slope signal.

The synchronization error is within ±3 mm during the entire motion. The comparison results of the three controllers are demonstrated in Figure 14. The curvilinear trend is familiar with the step signal. The LADRC-SC performs better than the others.

**Figure 15:**
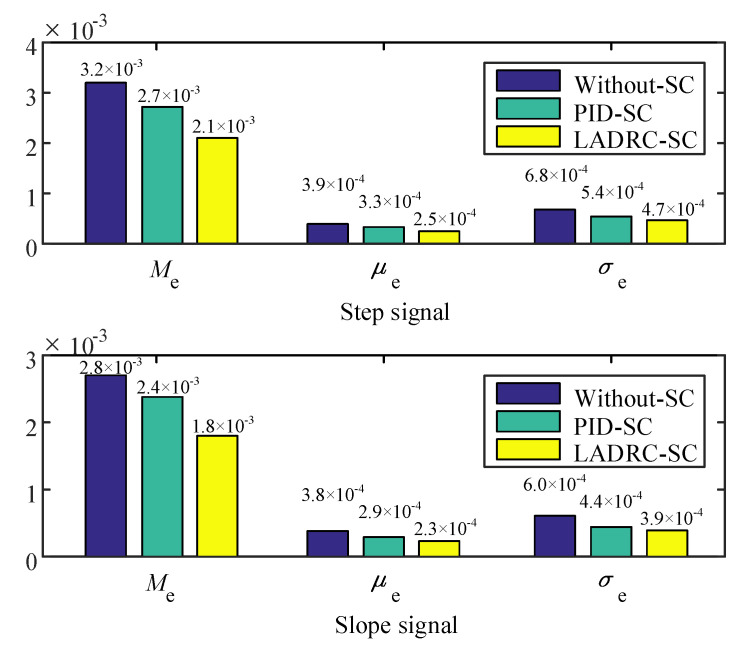
Performance indexes of different control schemes.

The synchronization error performance indexes of the two signals are displayed in Figure 15. It can be seen from Figure 15 that the three performance indexes of LADRC-SC are all the minimum under the step and slope input signals. When the step signal was input, the maximum absolute value $M_{e}$, average tracking error $\mu_{e}$, and standard deviation $\sigma_{e}$ of the LADRC-SC were reduced by 22.22%, 24.24%, and 12.96%, respectively, compared with the PID controller. Simultaneously, the reductions of the slope signal input were 25.00%, 20.69%, and 11.36%, respectively. Thus, according to the three performance indexes comparison results, the conclusion that LADRC-SC has the best performance can be drawn, and the effectiveness of the proposed method is verified.

## 6. Conclusions

This paper proposed a dual-hydraulic actuators synchronous control strategy to achieve high precision position synchronization control of a typical electro-hydraulic proportional position control system (EPPS) in the presence of unknown dead-zone nonlinearities. Forgetting factor least squares (FFLS) algorithm was employed to identify the model of the EPPS. Then, using model reference dead-zone inverse adaptive controller (MRIADC), dead-zone parameters were estimated by the adaptive law and compensated by the dead-zone inverse. Sinusoidal tracking experiments showed that the MRIADC could effectively compensate for the system dead-zone nonlinearity and improve the position tracking accuracy of hydraulic actuators within the range of ±2.5 mm. On the premise of ensuring the position tracking accuracy of each hydraulic actuator, an error-based LADRC synchronous controller (LADRC-SC) was introduced to improve the position synchronization control accuracy. A comparison with two other available controllers based on the same test platform was carried out. The position synchronization accuracies of the step signal and ramp signal were improved by 34.4% and 35.7%, respectively. 

## Figures and Tables

**Table 1:** Identification values of the model parameters.

Symbol	Value	Symbol	Value
${\hat{a}}_{11}$	−1.118	${\hat{a}}_{21}$	−1.003
${\hat{a}}_{12}$	0.1751	${\hat{a}}_{22}$	0.03041
${\hat{a}}_{13}$	−0.1332	${\hat{a}}_{23}$	−0.01021
${\hat{a}}_{14}$	−0.0761	${\hat{a}}_{24}$	−0.0172
${\hat{b}}_{11}$	$4.437\times 10^{-5}$	${\hat{b}}_{21}$	$5.795\times 10^{-5}$
${\hat{b}}_{12}$	$1.81\times 10^{-5}$	${\hat{b}}_{22}$	$1.504\times 10^{-6}$
${\hat{b}}_{13}$	$1.099\times 10^{-6}$	${\hat{b}}_{23}$	$-1.342\times 10^{-5}$
${\hat{b}}_{14}$	$-6.873\times 10^{-7}$	${\hat{b}}_{24}$	$3.025\times 10^{-5}$

**Table 2:** Hardware components specifications of the test platform.

Components	Type	Specifications	Values
AC motor	Y112M-4	Rotational speed	1440 rev/min
Hydraulic pump	10MCY14-1B	Displacement	10 L/rev
Proportional directional valve	HD-4WRA6E07-2X/G24Z4	Rated flow	30 L/min
Hydraulic actuator	YW140LLBDCS-200D0AE	Piston diameter	40 mm
		Piston rod diameter	25 mm
Displacement sensor	KTC-200	Linearity	0.05 mm
S7-300 PLC	6ES7315-2AH14-0AB0	-	-
S7-321 DI	6ES7321-1BH02-0AA0	Number of input channels	16
S7-322 DO	6ES7322-1HH01-0AA0	Number of output channels	16
A/D card	Advantech PCI-1710	Resolution	12-bit
		Maximum sampling rate	100 kHz
D/A card	Advantech PCI-1723	Resolution	16-bit
IPC	Advantech IPC-7132	-	-
